# Root Causes of Appalachia’s Deaths of Despair

**DOI:** 10.13023/jah.0102.01

**Published:** 2019-07-06

**Authors:** F. Douglas Scutchfield

**Keywords:** Appalachia, deaths, despair, life expectancy, drug overdose, suicide, alcohol use, excess mortality

## Abstract

The U.S. is experiencing a decline in life expectancy, particularly among rural white males in their most productive years. Appalachia is disproportionally represented in mortality rates, accounting for 30% of the U.S. population, but 50% of the excess mortality attributed to the “deaths of despair”: drug overdose, suicide, and alcoholic cirrhosis. A substantial proportion of that excess mortality is related to the current opioid crisis we are experiencing. We have data on evidence-based solutions to the treatment of addiction, but little information on prevention of addiction as well as the other deaths of despair, likely with the same etiologic agent. We must focus on finding the root cause of the current epidemic, so that we can prevent this devastating mortality.

This issue of the journal contains an article by Meit and colleagues that draws attention to the deaths of despair in Appalachia.^1^ It is one of two articles we have published that reflect on the rising mortality in the U.S., a substantial portion of which occurs in Appalachia. The notion of deaths of despair was developed by Case and Deaton based on their finding of an increasing mortality rate among white males in the midlife-age range from three primary causes of death: opioid overdose (likely linked to Fentanyl added to the opioid); suicide; and alcoholic cirrhosis of the liver.^2^ Meit points out that while this trend is occurring nationally, it is especially prominent in Appalachia, where, since 2000, the curves showing the mortality rates for these diseases have diverged. In the most recent data, rates in Appalachia are now 45% higher than the U.S. Meit et al. point out that these rates are highest in rural men, aged 45 to 54, in central and north central Appalachia and in distressed Appalachia counties, as defined by the Appalachia Regional Commission. Meit draws attention to the current opioid overdose mortality increase in Appalachia as one of the most concerning metrics in his discussion of deaths of despair in Appalachia.

In our first issue, Woolf and his colleagues^3^ used similar statistics that demonstrate the divergence of mortality in Appalachia compared to the nation and to Organization for Economic Cooperation and Development countries (OECD). Their data show that the U.S. is experiencing a decline in life expectancy that began in the 1980s, a finding that is not the case in OECD countries, which continue to experience a rise in life expectancy. This is a damning finding considering the amount of money the U.S. expends on medical care as opposed to comparable OECD countries. While Woolf et al highlight the deaths of despair and their contribution to this trend, they also make the point that mortality rates for cardiovascular, digestive, endocrine, and neurologic diseases are also rising in Appalachia. Woolf el al. also points out that while Appalachia contains about 30% of the U.S. population, its premature mortality is responsible for nearly 50% of the premature deaths in the U.S. The case of Appalachia substantially influences national trends!

Both Woolf 3 and Meit^1^ are struck by the contribution of opioid overdose deaths to this rising mortality and the deaths of despair. But as Woolf points out, the perverse rise in mortality is pervasive, with increases in mortality being broad-based, a finding suggesting underlying systemic etiologies of this trend. We currently have focused our attention and resources on the contemporary epidemic of opioids and on dealing with overdose deaths and the treatment of drug abuse. While salutary, this strategy does not address the etiology of drug abuse or allow us to identify the underlying cause of addiction so that addiction may be prevented before we are forced to deal with its consequences.

I applaud the attempt to more effectively identify and deal with the treatment of drug abuse and interventions to decrease the deaths due to overdose of drugs. We have evidence-driven approaches to both, including medically-assisted treatment of opioid abuse and pervasive availability of Naloxone to treat overdoses of opioids.^4^,^5^ There is no question that we should support and provide therapy, lifesaving in many circumstances, for people who are addicted. But this is not going to solve the problem. Just as we can’t incarcerate ourselves out of this epidemic, we cannot treat ourselves out of this epidemic. A quick look at the literature, using PubMed, suggests that the evidence that allows us to deal with the primary prevention of drug abuse is minimal, at best. We must identify the root cause of drug abuse and addiction and the plagues that have come to us, increasing mortality rates across the board, particularly of our middle-aged, male, rural Appalachian population. We must identify evidence-based interventions that work to prevent the drug abuse, suicide, and alcoholism that lead to the deaths of despair. We must identify and practice evidence-driven approaches to primary prevention to deal with the root causes of this mortality trend and prevent it from occurring.

We do have some evidence about etiology. Woolf 3 points that the health problems appear to have a systemic etiology given its breadth and depth. He suggests that these systemic causes are derived from socioeconomic forces, loss of mining and manufacturing jobs, and lack of education, which contribute to unemployment, income inequities, the physical environment including housing problems, food insecurity, racism, and harmful health habits born of these underlying issues.^2^ Quinones, in his book *Dreamland*,^6^ thinks the etiology of our drug abuse problem is a loss of social connectiveness, what those of us in the trade call social capital. The impact of this decline in social capital is well described in Putnam’s classic book, *Bowling Alone*. Unfortunately, in his book, Putnam identifies the problem but offers no solution.^7^ The issue is how do we build social capital, particularly in a time of the pervasive decline in civility of our people and the continued perception of Appalachians as “other”?

It is likely that our efforts to identify and mitigate this opioid epidemic will identify policies, and not necessarily health policies, that would drive the potential solutions to this problem and the other deaths of despair as well. If it is systemic issues that stem from our national economic and social spending and programs, the fix will not be easy, as we are so polarized. However, while we must contend with the outcomes of the opioid epidemic this should not preclude our efforts to find the etiology of this major health issue and address the root causes of this and the other mortality increases in Appalachia. When we know the etiology then we can productively engage in prevention of this mortality increase. Money needs to be targeted at these two major issues—etiology and interventions—that must drive our policies and our programs. We need and must have the research to answer questions related to etiology and evidence-based interventions. We must, again, be the upstream investigators rather than the downstream service providers, or the problem will not be solved.
